# Prospects for Cell-Directed Curative Therapy of Phenylketonuria (PKU)

**DOI:** 10.1142/s2529732519400145

**Published:** 2019-12-12

**Authors:** Cary O. Harding

**Affiliations:** 1 Department of Molecular and Medical Genetics, Oregon Health & Science University, Mailstop L-103, 3181 Sam Jackson Park Rd., Portland, OR 97239, USA.

**Keywords:** Phenylketonuria, Phenylalanine Hydroxylase Deficiency, Phenylalanine, Hepatocyte Transplantation, Liver-Directed Gene Therapy, Gene Editing

## Abstract

Phenylketonuria (PKU) due to recessively inherited phenylalanine hydroxylase (PAH) deficiency is among the most common inborn errors of metabolism. Dietary therapy begun early in infancy prevents the major manifestations of the disease but shortcomings to treatment continue to exist including lifelong commitment to a complicated and unpalatable diet, poor adherence to diet in adolescence and adulthood, and consequently a range of unsatisfactory outcomes, including neuropsychiatric disorders, frequently develop. Novel treatments that do not strictly depend upon dietary protein restriction are actively sought. This review discusses the potential for and the limitations of permanently curative cell-directed treatment of PKU, including liver-directed gene therapy and gene editing, if initiated during early infancy. A fictional but realistic vignette of a family with a new baby girl recently diagnosed with PKU is presented. What is needed to permanently cure her?

## INTRODUCTION

The phone call came on a beautiful Thursday afternoon in April. Molly Lynn Campbell, just 5 days old, was sleeping soundly after just having breast fed when her Mother Rachael answered the call from Molly’s pediatrician.

‘Hello, Rachael? This is Dr. Forrest. I am calling to check up on you and Molly.’

‘We’re doing fine. She’s just gone down for a nap,’ Rachael stated hesitantly, sensing that there was more to this call than just a routine check.

‘Hmmm….I am also calling because I have concerning news from Molly’s newborn screening test results.’

After a long silent pause between them, she continued, ‘You do remember before you and Molly left the hospital that she had her heel poked and a blood sample taken on to a filter paper card when she was about 24 hours old?’

‘Yes, I recall that.’

‘The hospital sends that card out to laboratory where they test for many different inherited diseases. The laboratory called me today to tell me that Molly’s test is positive for PKU.’

Rachael called for her husband Rick to join her in the discussion.

‘Dr. Forrest, Rick is here too and I’m putting you on speaker phone if that’s OK. Can you please repeat that and explain what this means?’

‘Hello, Rick, Dr. Forrest here and I’m so sorry to disturb your afternoon, but the lab just called me with this result. Molly’s newborn screening test is positive for PKU, which stands for phenylketonuria. It’s a genetic disease that has to do with protein metabolism. I don’t know much about it. There is a special diet for it.’

Rachael and Rick stood in silence, each experiencing in their own individual way, the acute pain of losing their previously perfect child.

Breaking the silence, Dr. Forrest continued encouragingly, ‘Now, this is just a screening test. There is a chance that it is wrong. The first thing we need to do is draw more blood for additional testing and confirm that she really does have PKU. If that second test is positive, then we’ll refer you to the Metabolic Clinic at the University Hospital. They’re the experts on these conditions and they’ll start her on the appropriate treatment. I’ve spoken to one of their physicians who assures me that there isn’t anything life-threatening about PKU. There isn’t anything we need to do emergently. If she has PKU, they’ll take good care of her there. The first thing is to confirm whether she really has it or not. I have called in an order to our laboratory for the confirmatory testing. You can take Molly there tomorrow morning for the blood draw. The test is again sent out to another laboratory and it’ll take several days to get the results back. In the meantime, we don’t need to change her diet or do anything. We just need to wait until the test result come back in about a week.’

The following week while waiting for the test results, Rachael and Rick alternated between the joy of caring for their physically gorgeous baby girl with the agony, apprehension, anger, disbelief, dread, resignation, resolve, all of it, all at once that their beautiful Molly actually had this hidden disease that threatened her brain. And worst of all, they had given it to her! Rachael and Rick learned from Wikipedia that untreated PKU can lead to *severe intellectual disability, autism, seizures, behavioral problems, and mental disorders. Treatment included a diet low in foods that contain phenylalanine*, but what did that mean? Babies take a *special formula with a small amount of breast milk*. But what comes after that? And *diet should begin as soon as possible* but Molly is now 10 days old! What is happening to all of her brain cells while we wait to learn the results of this test?!! People who are diagnosed early and *maintain a strict diet can have normal health and a normal life span*. But what about their brain function? *Treatment should continue for life*. In one way, this is worse than cancer! It won’t kill you but you can’t cure it either. It’s with you for life. Why isn’t there a cure? Rick and Rachael are intrigued by a vague sentence in the Wikipedia entry that begs for more exploration, ‘*Gene therapy, while promising, requires a great deal more study*…‥’

## PHENYLKETONURIA, ITS CAUSES, CONTEMPORARY TREATMENT, AND OUTCOMES REVIEWED

**Figure 1.**
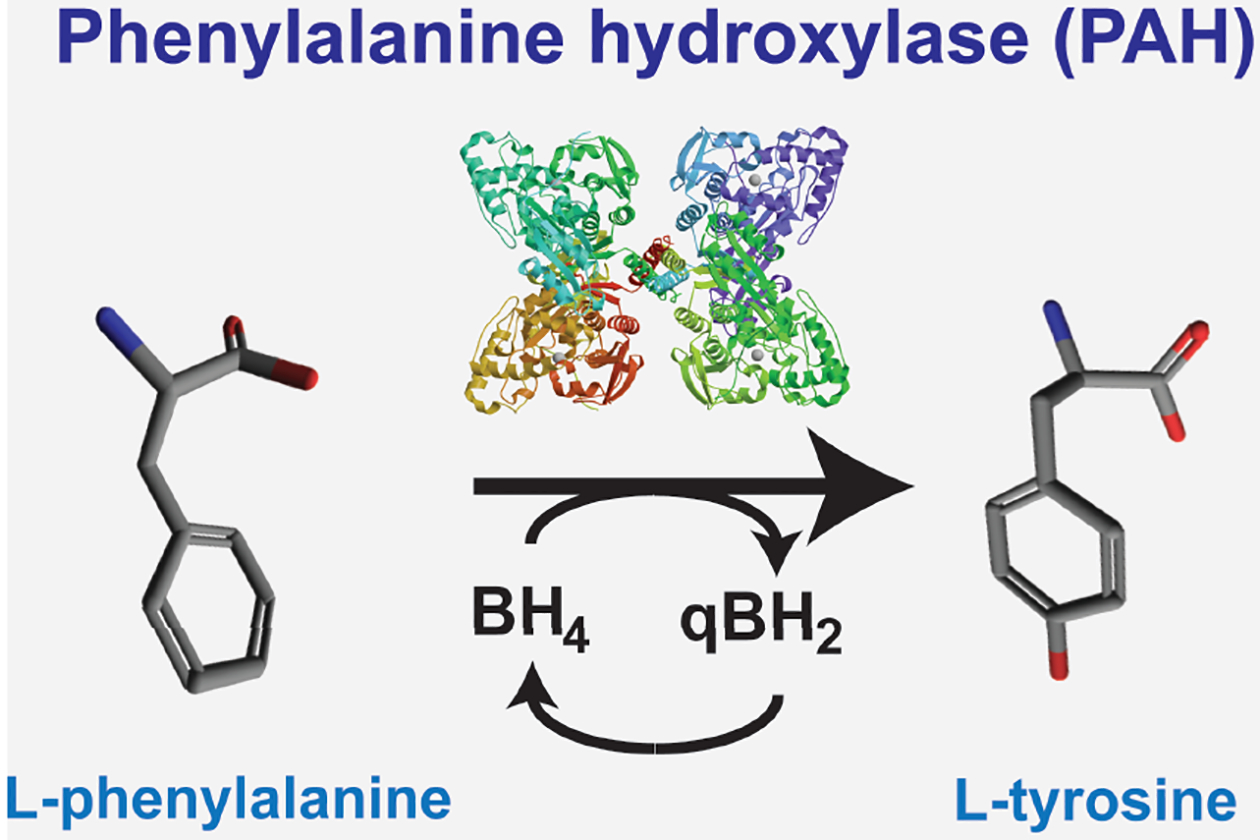
Phenylalanine hydroxylase (PAH) reaction and Phenylketonuria (PKU). Phenylalanine hydroxylase is a cytoplasmic iron and molecular oxygen-requiring homotetramer that catalyzes the conversion of L-phenylalanine to L-tyrosine in a reaction that utilizes the reduced pterin cofactor, tetrahydrobiopterin (BH_4_). The oxidized pterin, quinonoid dihydrobiopterin (qBH_2_), is enzymatically reduced back to BH_4_ and recycled to support continued Phe hydroxylation in liver. In recessively-inherited phenylketonuria (PKU), PAH activity is diminished or absent. PAH deficiency leads to accumulation of L-phenylalanine, relative L-tyrosine deficiency, and enzymatically catalyzed deamination of L-phenylalanine to the phenylketone, phenylpyruvic acid.

Phenylketonuria is the colloquial term for recessively-inherited deficiency of the enzyme phenylalanine hydroxylase (PAH) (Fig. 1) and refers to the chemical phenotype associated with the original description of the disease from 1934. The Norwegian physician, Asbjörn Fölling first described the urinary excretion of phenylpyruvic acid, a phenylketone, in two cognitively disabled siblings^1^, naming the disorder imbecillitas phenylpyrouvica, and later found several other individuals with a similar phenotype among institutionalized individuals in Oslo. He correctly surmised that the primary defect likely involved metabolism of the amino acid l-phenylalanine (Phe). The geneticist Lionel Penrose demonstrated that phenylpyruvate excretion increased further when dietary phenylalanine intake increased and he coined the term phenylketonuria^2^ to describe the disorder. PAH deficiency as the primary defect in PKU was not actually proven until almost 20 years later^3^.

Newborn screening is based upon the detection of elevated blood Phe^4^. Although the majority of infants with persistent hyperphenylalaninemia have PAH deficiency, in rare instances elevated blood Phe may also be caused inherited defects in the synthesis or recycling of tetrahydrobiopterin (BH_4_), a required cofactor for PAH activity (Fig. 1)^5^. Recently, a novel cause of hyperphenylalaninemia has been described: the deficiency of DNAJC12^6^, a chaperone protein required for the appropriate folding of PAH protein. So, if hyperphenylalaninemia persists in Molly beyond the initial newborn screen, then the next step in Molly’s evaluation will be to establish the precise molecular cause through sequencing of a panel of potentially causative genes including *Pah*, the gene encoding the enzyme PAH. Again, the majority of infants with hyperphenylalaninemia will be found to have mutations in *Pah*.

The mainstay of therapy for PAH deficiency is dietary restriction of Phe intake^7,8^ through restriction of dietary intact protein. Because Phe is an essential amino acid, the concentrations of Phe in blood and other tissues can be controlled to some degree through diet therapy, although this becomes increasingly difficult with age. In adults, less than 10% of dietary Phe in the typical diet is necessary for the maintenance of normal protein turnover; the remaining 90% must be hydroxylated to tyrosine. Because Phe hydroxylation is severely impaired in individuals with inherited or functional PAH deficiency, the majority of dietary Phe intake must be eliminated in order to prevent severe hyperphenylalaninemia. All meat, dairy products, standard breads and pastas must be completely avoided; some specific vegetables that are high in free Phe are also restricted. A combination of specialized medical formulas and low protein food products supplying sufficient energy, vitamins, micronutrients, and amino acids other than Phe must be consumed to maintain balanced nutrition. It is recommended that individuals with hyperphenylalaninemia maintain blood Phe concentrations at 120–360 μM^7,8^ (normal 60–120 μM) throughout the lifespan^9^, although there is not yet international agreement upon the specific treatment targets^10^.

A subset of individuals with PAH deficiency are responsive to treatment with sapropterin dihydrochloride, a synthetic form of the natural BH_4_ cofactor^11^. These individuals carry *Pah* genotypes that yield some residual PAH enzyme activity^12^ and typically exhibit milder hyperphenylalaninemia at baseline. Sapropterin likely acts as a chaperone to stabilize the mutant PAH protein^13^ leading to improved Phe tolerance and allowing a lessening of the dietary Phe restriction that the patients must follow. The proportion of PAH deficient patients who are responsive to sapropterin is between 20–50%^14,15^, but in the majority, some degree of dietary Phe restriction must be continued along with sapropterin therapy.

Recently, a novel enzyme substitution therapy for adults with PAH deficiency has been developed. Pegvaliase is a form of recombinantly-produced phenylalanine ammonia lyase from the cyanobacterium Anabaena variabilis. Administered by daily subcutaneous injection, pegvaliase circulates in blood and converts Phe to trans-cinnamic acid and ammonia. Several phase 1, 2, and 3 clinical trials have demonstrated the efficacy of pegvaliase in decreasing blood Phe concentration in adults, even down to the normal range, while liberalizing dietary Phe intake^16–19^. This revolutionary treatment approach is increasingly available in the US and Europe, yet still demands daily injections and is associated with immune-mediated hypersensitivity reactions in some individuals. It is not a cure.

Newborn screening and dietary treatment of hyperphenylalaninemia has been commonplace since the 1960’s, but the field had been permeated for some years by the unfortunate and unfounded perception that the problem of PKU had been completely solved. One can only surmise that this impression developed out of the need on the part of practitioners in the field to constantly champion and defend dietary treatment against naysayers and reluctant payers as dietary therapy was the only available effective treatment (prior to 2007) and was essential to prevent severe cognitive disability in affected infants. Early studies indicated that dietary Phe restriction begun during infancy in PAH deficiency dramatically improved the developmental outcomes of affected patients as compared to the expected natural history of the disease^20–26^, yet objections were raised against the adequacy of the evidence base supporting the efficacy of diet therapy. A large multicenter collaborative study was therefore designed to systematically study outcomes in infants with PAH deficiency detected through newborn screening^27^; the original goal was to compare outcomes in infants randomized to two different blood Phe targets (120–360 or 360–600 μM). Ultimately, it proved impossible to maintain sufficient dietary control to keep blood Phe precisely within the target ranges over time with the dietary tools available so the comparison between the two targets was unsuccessful. That said, the study did prove the efficacy of therapy in young children and established a direct relationship between chronic blood Phe control and cognitive outcome in children with PKU — dietary Phe restriction in PAH deficiency was proven to prevent the major manifestations of the untreated disease (severe cognitive disability, seizures, growth failure)^28^. Importantly, the study showed significantly higher IQ in children who remained on dietary Phe restriction through age 12 years in comparison to those in whom diet restrictions had been discontinued at age 6 years. A further, less well appreciated finding from that study was the comparison between 12 year old children with PAH deficiency on diet (with a treatment target of Phe = 120–600 μM) in comparison to their PAH sufficient siblings; the children with PAH deficiency exhibited a normal mean IQ of 100 but the mean IQ of their siblings was 10 points higher at 110, and the incidence of attention deficit and specific learning disabilities, particularly in visuospatial tasks, was significantly greater in children with PAH deficiency despite adequate dietary therapy for the time. Unfortunately, funding for the collaborative study was discontinued when the study essentially achieved its goal of proving the efficacy of dietary therapy for infants and children. Although a follow up of some of the original participants in the collaborative study was published some years later and demonstrated significant deterioration in cognitive function in those who had lost dietary control (with a high incidence of educational, social, and vocational dysfunction)^29^, no large-scale systematic evaluation of health, cognitive, or psychiatric outcomes in adolescents or adults with hyperphenylalaninemia exists. Furthermore, no comparison of the various treatment modalities available for adults has been conducted. Still, we know that outcomes remain imperfect in many adolescents and adults.

- The incidence of inattention and learning disabilities in children with PAH deficiency treated with dietary Phe restriction remains greater than in their PAH sufficient siblings and in the general population^28^.
- Adherence to dietary therapy in adolescents and adults is poor with up to 85–90% of patients exhibiting blood Phe concentrations above target levels^30,31^.
- Adults with chronic hyperphenylalaninemia exhibit a high incidence of psychiatric symptoms including anxiety and depression^32,33^ and upon detailed neuropsychological assessment exhibit inattention and impaired executive function^34^.
- Hyperphenylalaninemia in individuals with PAH deficiency is associated with diffuse abnormalities of brain white matter microstructural integrity visible by diffusion tensor imaging and concomitantly with impaired executive function on neuropsychological tests^35,36^.
- Many adolescents and adults with elevated blood Phe are hyperreflexic and many exhibit intention tremor^37^. Severe white matter damage visible by MR imaging associated with tone abnormalities, impaired balance and gait, and even seizures can occur in some adults with chronic hyperphenylalaninemia^38^.
- Elevated blood Phe during pregnancy causes severe teratogenic effects upon the fetus, the so-called maternal PKU syndrome^39^. Elevated maternal blood Phe crosses the placenta and interferes with early fetal development leading to microcephaly, facial dysmorphism, global developmental disability and congenital heart disease in a substantial percentage of infants.
- The long term health consequences of dietary therapy are a concern with questions regarding vitamin and micronutrient status^40–42^, and the effects of hyperphenylalaninemia or diet therapy upon kidney^43^ and bone health^44,45^.

All of these concerns provide a compelling argument for the development of novel therapies for hyperphenylalaninemia that do not strictly depend upon dietary Phe restriction. Patients with PAH deficiency and their families have expressed, through a survey conducted by the National PKU Alliance (NPKUA), their overwhelming desire for the development of novel treatments^46^.

## POTENTIAL CELL-DIRECTED CURATIVE THERAPIES FOR PKU

PAH is predominantly expressed in liver, specifically in the cytoplasm of hepatocytes, so the aim of a truly curative therapy is to yield a permanent population of PAH-expressing hepatocytes in the individual with PKU that is sufficient to metabolize the daily phenylalanine load from both endogenous protein turnover and from unrestricted dietary protein intake. PAH is also expressed in kidney^47^, but it is known that restoration of liver PAH is sufficient to completely correct hyperphenylalaninemia. A child with PKU has undergone orthotopic liver transplantation for reasons unrelated to PKU^48^ and this was found to completely correct his dietary Phe tolerance. Although this procedure would be curative, the scarcity of donor livers precludes their routine utilization in a disorder that is not life threatening. Instead, preclinical investigations over many years have focused upon two potential avenues of treatment: cell transplantation to provide a population of PAH positive cells or alternatively, gene therapy to restore PAH activity in the patients’ own cells. Fortunately, a murine model of human PAH deficiency, the *Pah*^*enu2/enu2*^ mouse^49^, which harbors a missense mutation in the *Pah* gene^50^ and recapitulates the major features of human PKU, has been available for use as a test bed for novel treatments.

### How many PAH+ hepatocytes are required to correct hyperphenylalaninemia?

Humans and mice that are heterozygous for mutations in Pah have 50% or less liver PAH activity and exhibit blood Phe concentrations in the normal range on typical dietary protein intake; this fact suggests that it will not be necessary to fully restore cellular PAH activity to 100% following liver-directed gene transfer in order to affect blood Phe concentrations. In these instances, however, PAH activity is spread among all hepatocytes in the liver. Transplantation of a small population of wild type hepatocytes or supraphysiologic expression of PAH activity in a small hepatocyte population following gene transfer is not guaranteed to have the same effect upon Phe clearance. In fact, hepatocyte transplantation experiments into *Pah*^*enu2/enu2*^ mice have demonstrated that PAH activity must be distributed across a minimum number of hepatocytes otherwise Phe flux becomes limited^51,52^. Blood Phe concentration was completely corrected in *Pah*^*enu2/enu2*^ mice that had been repopulated with as few as 10% wild type hepatocytes expressing 100% PAH activity but remain mildly elevated in mice that achieved only 5–10% repopulation^51^. Surprisingly, identical results were demonstrated in mice transplanted with hepatocytes from heterozygous *Pah*^*enu2/*+^ mice, which express less than 50% PAH activity^52^. This result suggested that at low repopulation frequency with PAH+ hepatocytes, the absolute cell number is primarily limiting Phe clearance rather than the total PAH enzyme activity. So regardless of the treatment approach taken, cell transplantation or gene therapy, *complete therapeutic success will likely require achieving a stable population of least 10% PAH-expressing hepatocytes in the liver of an individual with PKU*.

## THERAPEUTIC LIVER REPOPULATION (TLR) FOLLOWING CELL TRANSPLANTATION

TLR following cell transplantation is an alternate treatment approach to whole organ transplantation^53^. Liver is unique among solid organs in its capability to completely regenerate itself after injury. TLR takes advantage of this regenerative potential and aims to replace diseased liver with fully functional hepatocytes. Successful TLR is dependent upon a stimulus for liver regeneration at the time of hepatocyte transplantation and ideally a selective growth advantage for the donor cells over the native hepatocytes. Transplantation of wild type hepatocytes into healthy liver of adult mice unfortunately yields little repopulation, most likely due to the low level of ongoing hepatocyte turnover in the normal adult liver. The latter likely leads to insufficient driving force for the growth of transplanted cells. This has led many investigators to introduce liver damaging interventions in order to induce engraftment of exogenous hepatocytes, including hepatectomy, irradiation, or the use of alkaloids and other chemicals to deplete endogenous liver tissue. A single trial of hepatocyte transplant into an adult with PKU has been carried out at the University of Pittsburgh (IJ Fox, personal communication). In this patient, focal gamma irradiation of the left liver lobe was employed prior to delivery of donor hepatocytes to generate both a regenerative stimulus but also to block mitosis of the native hepatocytes and allow preferential proliferation from the donor hepatocytes. Even with this preparative regimen, only minimal liver repopulation and a modest decrease in blood Phe concentration was achieved.

Transplantation into neonatal liver, in which the hepatocyte population is rapidly expanding during normal growth, offers greater promise for successful TLR. Evidence for the active function of transplanted human hepatocytes in infants was first obtained in a patient with Crigler–Najjar syndrome type I (UDP-glucuronosyltransferase 1A1; UGT1A1 deficiency), in whom there was a fifty percent reduction of serum bilirubin levels and reconstitution of 5% of hepatic UGT1A1 activity following a single hepatocyte transplantation. This study established that long-term engraftment and function of transplanted allogeneic hepatocytes could be accomplished^54^. Successful allogeneic TLR was subsequently reported in ornithine transcarbamylase (OTC) deficiency (a urea cycle disorder)^54–56^. In a recent clinical trial of hepatocyte transplantation in several infants with urea cycle disorders, repeated hepatocyte infusions via the umbilical vein were performed over the first two weeks of life in order to gradually build up a physiologically relevant population of wild type cells^57^. This led to transient improvement in biochemical and clinical parameters in some but not all patients. However, the investigators struggled to maintain sufficient immunosuppression, which was necessary as the donor cells were allogeneic. All subjects ultimately received orthotopic whole liver transplants and upon detailed examination of the explanted livers, little evidence of the transplanted hepatocytes infused during the neonatal period could be found. The investigators concluded that the donor hepatocytes had most likely been eliminated by the host immune system. To date, a neonatal hepatocyte transplantation trial has not occurred in PKU.

Cell sources other than fully differentiated adult hepatocytes have been suggested as possible donor cell sources, such as induced pluripotent stem cells or even embryonic stem cells, which would then hopefully differentiate into fully functional hepatocytes. Human amnion epithelial cells (hAEC), the cells that form the epithelial layer on the external surface of the human placenta, are an interesting potential source of cells for TLR^58^. These unique cells are pluripotent, being capable of differentiating into several different cells types including hepatocytes, but most interestingly, hAEC express a cell surface antigen, HLA-G, that leads to immunologic tolerance of the cells in the recipient and absence of any need for immunosuppression following transplantation.

## GENE THERAPY

**Figure 2.**
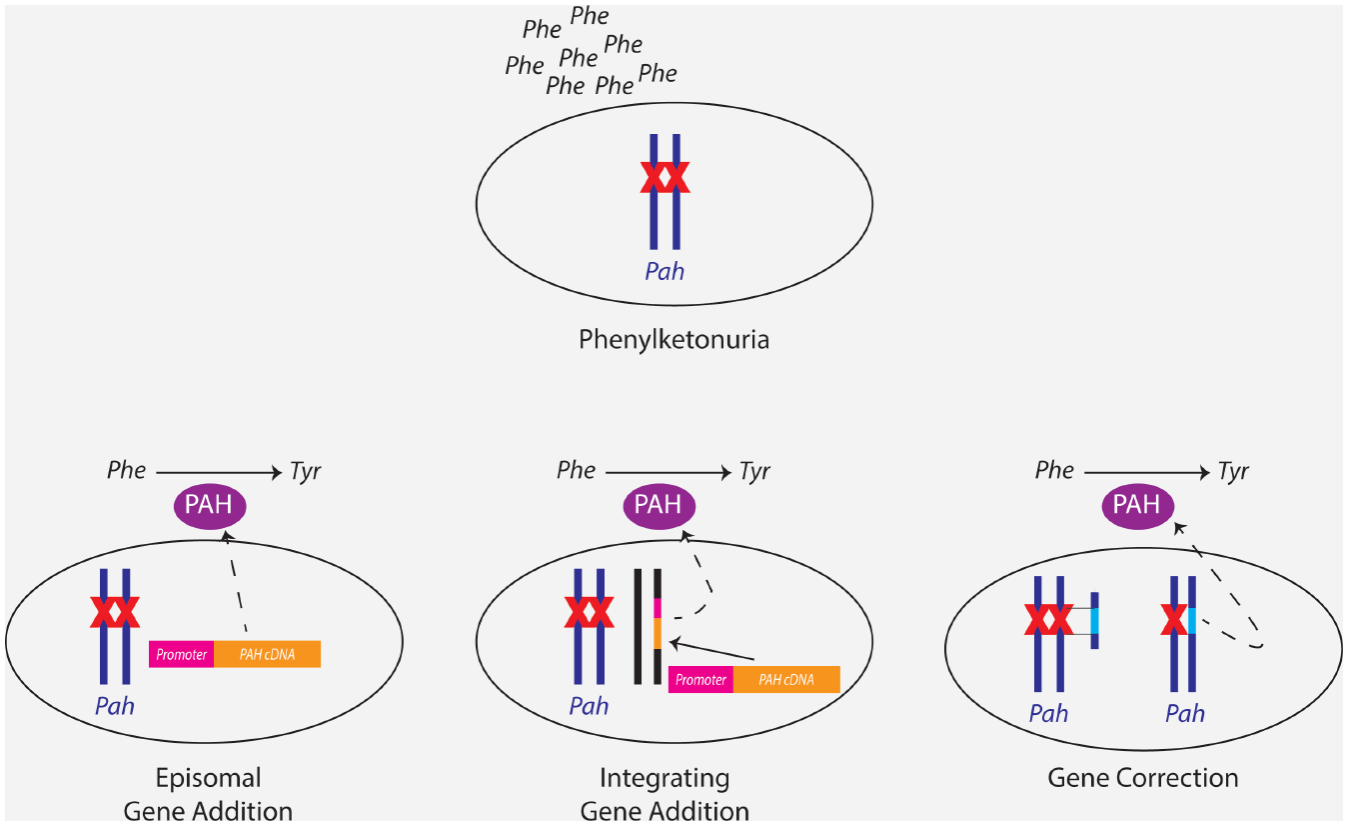
Gene therapy for PKU. Phenylketonuira (PKU) is caused by recessively-inherited mutations (X) in the Pah gene leading to PAH enzyme deficiency and Phe accumulation. Three gene therapeutic approaches are used to restore cellular PAH enzyme expression and reduce Phe. First, in episomal gene addition, a gene therapy vector expressing PAH from a PAH cDNA driven by a promoter is introduced into the PAH-deficient cell. The vector genome resides autonomously within the host cell nucleus as an episome that is not integrated into the host cell DNA. In integrating gene addition, the vector genome encoding the PAH expression cassette physically integrates somewhere in the host cell genome, not necessarily into the chromosome carrying the native Pah gene. In neither of these two approaches is the native Pah gene altered. Lastly, in gene correction, the disease-causing Pah gene mutation is corrected back to the wild type sequence to restore PAH expression.

The Wikipedia entry on PKU ended with a less than definitive proclamation about the potential for gene therapy as a treatment for the disease. So what is the current status of this treatment approach? By definition, gene therapy is the use of genes, typically DNA, as a drug to treat a disease. For the treatment of genetic disorders, gene therapy comes in two specific flavors, gene addition and gene correction (Fig. 2). In gene addition, an intact copy of a functional gene is added to the diseased tissue or organism; the mutant gene present in the patient is not altered by this approach. This treatment method is appropriate for recessively-inherited disorders, such as PKU, where disease pathogenesis is caused by insufficiency of a specific gene product. Addition of a functional gene to produce the gene product will cure the disease. The method will not typically work for dominantly inherited disorders in which disease pathogenesis is caused by the presence of a mutant gene product; simple addition of normal gene product without elimination of the mutant protein will not ameliorate the disease. There is a further distinction among gene addition methods in whether the approach leads to integration of the added genetic material into the host genome or whether the added gene remains as a non-integrated episome in the nucleus of the host cell. In gene correction, the mutation within the disease gene of the patient is physically altered, leading to restoration of the wild type sequence, and production of the normal gene product. This approach will work for either recessive or dominant disorders but is typically more difficult to achieve. A complete review of the field of gene therapy is certainly beyond the scope of this article; the focus will remain upon those specific milestone experiments pertaining to treatment of PKU.

### Episomal gene addition approaches to the treatment of PKU.

**Figure 3.**
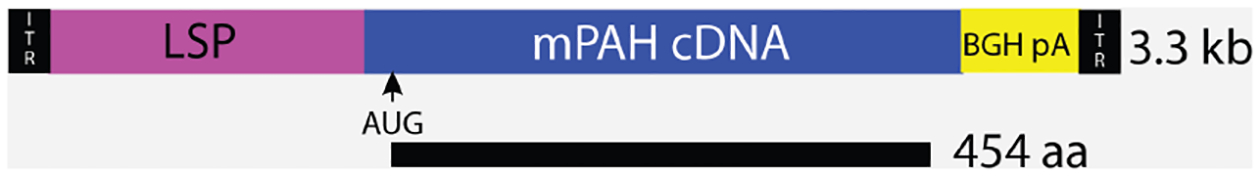
Representative recombinant AAV vector genome for liver-directed gene therapy for PKU. Diagram of a typical recombinant AAV vector genome designed for liver-directed gene therapy in *Pah*^*enu2/enu2*^ mice. Expression from the murine PAH complementary cDNA (cDNA) is driven by a strong Liver Specific Promoter (LSP). A canonical polyadenylation signal from the bovine growth hormone gene (BGH pA) is also required for appropriate expression. The only viral sequences retained the in genome are the two AAV intermediate terminal repeats (ITRs) flanking the expression cassette; these sequences are necessary for packaging of the genome into viral capsid proteins to form infectious viral particles and for the appropriate intracellular processing of the genomes following vector administration. The translation start site (AUG) is depicted at the 5’ end of the cDNA; translation initiated here yields a functional 454 amino acid PAH monomer that then spontaneously forms enzymatically active tetramers.

Several investigators have investigated the feasibility of gene addition for the treatment of PKU in preclinical experiments. Most experiments have exploited nature’s best gene delivery vehicles, various types of viruses, to deliver a normal *Pah* gene into liver of hyperphenylalaninemic *Pah*^*enu2/enu2*^ mice (reviewed in^59^), but a few labs have also exploited non-viral methods of delivering *Pah* into liver^60^. In the viral approaches, the majority of the viral sequences from the virus genome are replaced by the therapeutic gene (*Pah*), actually the Pah cDNA. Minimally, a strong promoter placed upstream and a polyadenylation signal downstream of the cDNA are necessary to drive tissue specific expression (Fig. 3). This recombinant genome is then transfected into the requisite cell line in culture where it is packaged *in vitro* with viral capsid proteins to form infectious but replication incompetent viral particles. The recombinant viral particles are purified, quantified and then administered to the target organism where ideally they infect the target organ (liver in the case of PKU) and direct the expression of the therapeutic gene product.

Two independent studies have employed recombinant adenovirus vectors, based upon a virus that causes the common cold, directed toward liver and demonstrated transient correction of serum Phe in *Pah*^*enu2/enu2*^ mice *in vivo*^61,62^. However, the effects were temporary because the virus elicited a profound host immune response that eliminated the vector transduced hepatocytes. A second administration of adenoviral vector had no effect because of anti-adenoviral antibodies now present in the treated animal. The inflammatory response induced following recombinant adenovirus administration in the mice presaged the inflammatory response that led to the death of an 18 year old male subject in a liver-directed adenovirus-mediated gene therapy trial for ornithine transcarbamylase (OTC) deficiency^63^.

Recombinant adeno-associated virus (rAAV) is currently the favored vector system for safe and effective liver-directed gene addition, with active clinical trials ongoing for adults with Hemophilia A or B, OTC deficiency, glycogen storage disease type 1A, acute intermittent porphyria, Crigler-Najjar syndrome, and others^64^. rAAV-mediated liver-directed gene therapy for PKU has been explored preclinically by several investigators using the *Pah*^*enu2/enu2*^ mouse model for nearly twenty years. AAV is a non-pathogenic parvovirus that is biologically unrelated to adenovirus; it is called adeno-associated simply because it requires the presence of adenovirus to replicate. Several different laboratories have demonstrated successful treatment of *Pah*^*enu2enu2*^ mice using various flavors of rAAV with differing viral capsid serotypes^65–70^. Administration of rAAV pseudotyped with serotype 8 capsid has consistently yielded the most robust correction of blood Phe in mice; delivery of 10^11^–10^12^ vector genomes of rAAV to adult *Pah*^*enu2/enu2*^ mice by intravenous injection is associated with rapid decrease in blood Phe down to normal concentrations. The recombinant AAV genomes do not integrate into the host liver genome but rather multiple AAV genomes combine within the hepatocyte nucleus to form circular head-to-tail episomes as the predominant PAH-expressing molecular species. rAAV treatment of the mice does not cause any notable adverse effects, and the safety profile has been outstanding in multiple human clinical trials for diseases other than PKU. Because of the accumulating human safety data and the body of preclinical efficacy evidence, human clinical trials of rAAV-mediated liver-directed gene therapy using different AAV serotypes are now planned in adults with PKU.

rAAV genomes predominantly remain episomal and only rarely integrate into the liver genome, as discussed. Episomes are lost and therapeutic gene expression diminished if hepatocyte division occurs. In preclinical systems, rAAV-mediated therapeutic gene expression in liver is lost following partial hepatectomy. Hepatocytes in the remaining liver rapidly divide to regenerate the needed liver mass, and during that regeneration, all rAAV episomes and therapeutic gene expression are lost. Also, therapeutic gene expression following rAAV administration to neonatal animals is short lived typically, as rapid liver growth and hepatocyte division is again associated with quick loss of rAAV episomes^71,72^. So unfortunately, standard liver-directed gene addition approaches that only leave episomal genomes in hepatocytes will not be a viable treatment for neonates with PKU.

## THE PROMISE OF GENE EDITING

A truly permanent gene therapeutic solution will require that the hepatocyte genome of the neonate with PKU be permanently altered so that the genetic alteration will be replicated and will persist in daughter cells even while treated hepatocytes undergo cell division during normal liver growth or during the process of liver regeneration following some toxic insult later in life. Two approaches (Fig. 2) are possible: a variation of *gene addition* in which a complete expression package with Pah cDNA and promoter are permanently integrated somewhere into the liver genome, not necessarily into the *Pah* gene, or *gene correction* in which the patient’s specific disease-causing mutation is corrected back to the normal sequence leading to restoration of PAH expression from the *Pah* gene. In either case, the hepatocyte gains permanent functional PAH expression. The latter approach has the theoretical advantage that PAH expression would be directed by the native *Pah* gene promoter and therefore regulated naturally by those influences that control native PAH expression. Gene expression from the gene addition method would just be constitutively activated at all times. Current experimental results fortunately do not suggest any major adverse effects from constitutive PAH expression in liver. The major drawback of the gene correction approach is the likely need to design reagents specific to every different mutation, and therefore a multiplicity of different gene therapy vectors would need to exist to treat the entire spectrum of PKU patients whereas a single vector system would be needed to deliver the PAH expression construct for gene addition.

### Integrating gene addition.

Wild type adeno-associated virus latently infects its host cell by integrating its genome into a specific site in the human chromosome, but unfortunately recombinant AAV vectors only very rarely integrate, so rAAV cannot be relied upon for effective permanent integrated gene addition. Recombinant gene therapy vectors based upon the human immunodeficiency virus (HIV), so-called lentivirus vectors, are much more efficient at permanent integration into the host cell. Because hematopoietic cells are the natural target of lentiviral infection, lentivirus-mediated gene transfer has become a favored method for integrating hematopoietic stem cell-directed gene therapy. However, lentivirus will infect liver, and there have been recent preclinical attempts at using lentivirus vectors to achieve permanent gene addition in liver^73^. Currently, lentivirus vector injection leads to permanent integrations in 1–2% of hepatocytes, a number that is insufficient to treat PKU, but investigators are working to develop methods that will provide hepatocytes harboring lentivirus integrations with a selective growth advantage to allow expansion of this population of hepatocytes to physiologic relevance.

### Gene correction.

**Figure 4.**
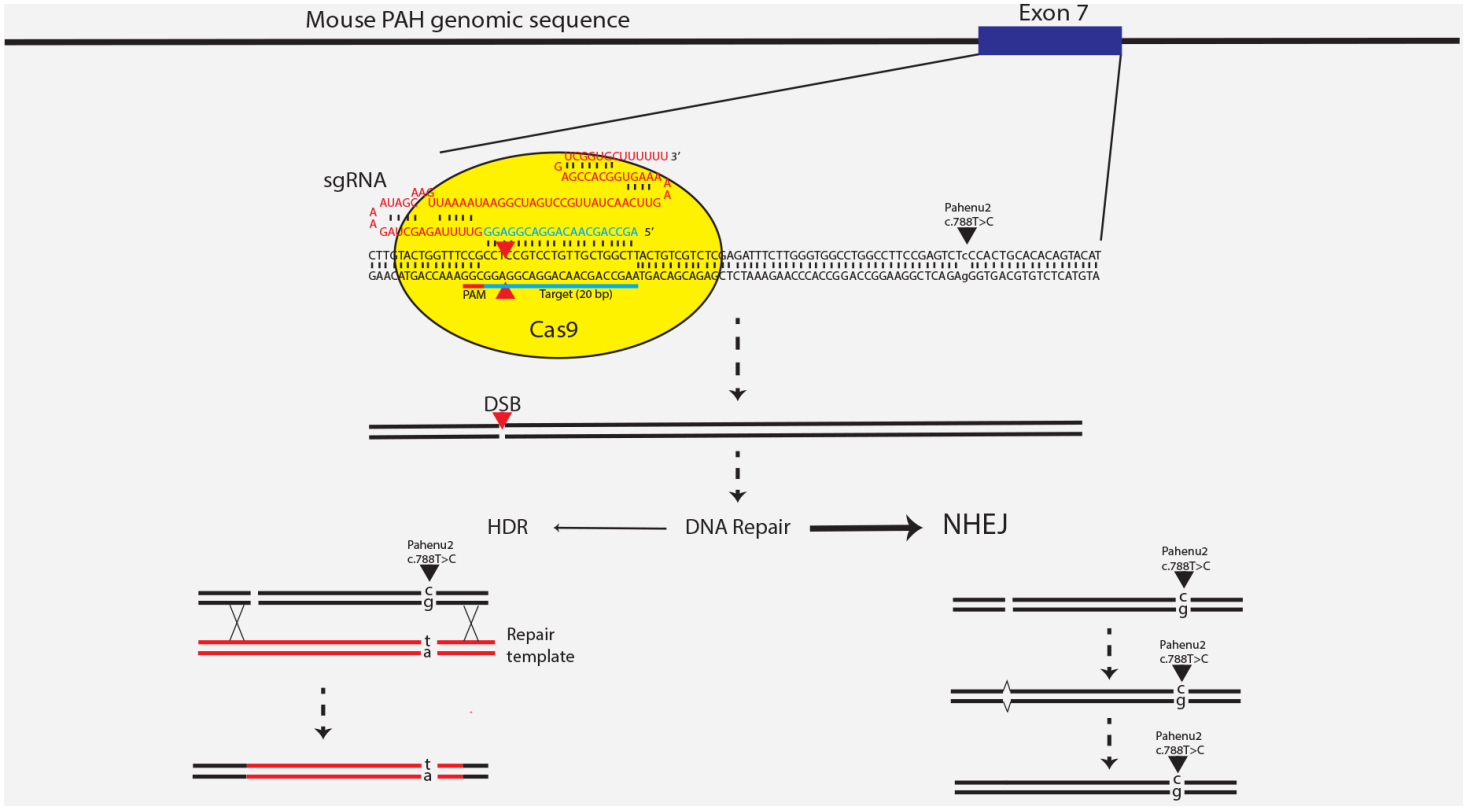
Site specific CRISPR/Cas9-mediated double strand breakage in the Pah gene and the critical choice of DNA repair pathway for editing of the *Pah*^*enu2*^ mutation. We designed a CRISPR/Cas9-mediated strategy to attempt to repair the *Pah*^*enu2*^ mutation in PKU mice. The *Pah*^*enu2*^ mutation is a T to C change at nucleotide position 788 (relative to the translation start site of the Pah cDNA) residing in exon 7 of the Pah gene. We designed a single guide RNA (sgRNA) that directs binding of the Cas9 endonuclease to a position just upstream from the *Pah*^*enu2*^ mutation site. Cas9 induces a double strand break (DSB) at this specific location. Competing innate cellular DNA repair mechanisms then seek to repair the DSB. The predominant repair mechanism is Non-Homologous End Joining (NHEJ), which can either perfectly repair the DSB yielding the original unaltered DNA sequence or may introduce small deletions or duplications (indels) further disrupting the gene. Neither of these events results in restoration of a functional Pah gene. Alternatively, in the presence of a double stranded DNA repair template that harbors the wild type sequence at the site of the targeted mutation, a small percentage of genomes may be repaired through Homology Directed Repair (HDR). In successful HDR, a segment of the chromosome harboring the DSB and the *Pah*^*enu2*^ mutation is replaced by the repair template sequence leading to correction of the mutation and restoration of PAH expression.

Gene correction, that is correction of the patient’s specific mutation back to the normal sequence, is an attractive approach to the treatment of genetic disease. CRISPR/Cas9 technology is a rapidly developing and widely adopted strategy for genome editing applications including gene correction in animal models of human disease (reviewed in^74^). The naturally occurring CRISPR (Clustered Regularly Interspaced Short Palindromic Repeats)/Cas (CRISP-associated gene) system is a key component of microbial adaptive immunity which bacteria use to protect themselves from viral invasion. The system employs an RNA-guided endonuclease (Cas9) to cleave specific CRISPR sequences in bacteriophage genomes, but this system has been adapted to also cleave mammalian DNA^75,76^. Once Cas9 induces a double strand break at a specific genomic site, the break can be repaired through one of two DNA repair mechanisms operating endogenously within the host mammalian cell, either NHEJ or less frequently homology directed repair (HDR) (Fig. 4). NHEJ can lead to perfect repair of the DSB back to the original sequence but may also result in a small deletion at the site of the initial DSB and cause the target gene to remain disrupted even after gene repair. This technique has been used to develop targeted knockout animal models. Alternatively, if an intact copy of genomic DNA with homology to sequences flanking the DSB is supplied along with the CRISPR/Cas9 machinery, this repair template can be integrated through HDR into the region of the DSB leading to gene correction. Due to the packaging size limit of rAAV, the necessary CRISPR/Cas9 reagents and repair template must be delivered in two separate rAAV vectors. Unfortunately, all things being equal, HDR with the repair template, which is the desired event necessary to correct disease, occurs much less frequently than NHEJ. Thus, when CRISPR/Cas9 was applied to adult mouse models for ornithine transcarbamylase deficiency, hereditary tyrosinemia, and hemophilia B, repair rates were extremely low, at 2%, 0.4%, and 1%, respectively^77–79^. Repair rates due to HDR in liver are higher if CRISPR/Cas9 reagents are administered to neonatal mice, reaching about 10% in neonatal OTC-deficient mice^80^. In a recently published experiment using CRISPR/Cas9 reagents delivered via rAAV serotype 8 vectors to neonatal *Pah*^*enu2/enu2*^ mice^81^, we found NHEJ predominance with repair of Cas9-mediated DSB back to the original mutant sequence. We achieved only about 1% HDR repair unless we also treated the mice with vanillin, a pharmacologic NHEJ inhibitor. In mice treated with both CRISPR/Cas9 reagents and vanillin, the frequency of HDR repair increased to 13% on average; this restored liver PAH activity to approximately 10% of normal and led to partial but incomplete correction of blood Phe concentration. Although this is an initially promising result, methods to further increase the frequency of HDR in preference to NHEJ must be developed, and the safety of the approach with regards to off-target DSB must be further examined, before CRISPR/Cas9-mediated liver-direct gene editing could be considered for human clinical trial.

Recently, novel CRISPR/Cas9-based genome editing tools that enable direct single base substitutions without introducing DSB have been developed and employed to treat animal models of genetic disease. These base editor systems are chimeric proteins consisting of endonuclease-deficient Cas9 fused to a catalytic domain from either a cytidine or adenine deaminase. The Cas9 protein along with the guide RNA allows targeting of the complex to the mutation site of interest but the Cas9 is catalytically dead and unable to create a DSB. Rather, the deaminase activity alters the base at the mutation site and ultimately allows a base substitution. The cytidine base editor deaminates a cytosine, creating uracil; during subsequent DNA replication, uracil is read as a thymine and the C∙G base pair has been successfully converted to T∙A^82^. Adenine base editors enable single A∙T to G∙C base pair conversions via inosine intermediates^83^. In a recent study, a cytidine base editor was employed to correct the c.788T>C missense mutation in the *Pah*^*enu2/enu2*^ mouse model back to thymine^84^. rAAV-mediated delivery of cytidine base editor into liver resulted in about 20% repair of *Pah*^*enu2*^ alleles, and a complete reduction of blood phenylalanine to physiological levels. These data suggest that the base editors have great potential for treatment of a subset of PKU patients with targetable T to C, A to G, G to A, or C to T mutations. Very recently, the laboratory of David Liu has developed a Cas9 nickase variant tethered to reverse transcriptase that is capable of utilizing an extended guide RNA to not only localize the enzyme to a mutation target but to also, using the extended guide as a template, transcribe in the correct DNA sequence in place of the targeted mutant sequence^85^. This so-called Prime Editing method is capable of editing all twelve types of point mutations as well as small deletions and insertions at significant frequency at least in cultured cells.

The major drawback of any of the site specific editing methods is that a different editing reagent design is required for each specific mutation, so that essentially each patient, or at least patients sharing the same mutation, will require their own custom gene therapeutic agent. Additionally, much effort remains prior to clinical use of these gene editing methods to evaluate the frequency of off target DNA changes elsewhere in the genome as these are potentially deleterious.

## CONCLUSION

So how do we cure PKU in Molly? A method that permanently restores a population of PAH-expressing hepatocytes that make up at least 10% or more of the liver and that are propagated as she grows is the holy grail for cell-directed PKU therapies in a neonate. Contemporary cell transplantation methodology has struggled to deliver sufficient cell numbers, to provide a selective growth advantage for the donor cells, or to prevent their immune rejection. Episomal gene addition would definitely only provide temporary treatment if applied to neonates. The integration frequency achieved by currently available integrating gene addition methods is too low to be physiologically relevant unless a method for establishing a selective growth advantage for hepatocytes with integrations can be achieved. Gene correction by HDR following CRISPR/Cas9-mediated gene cleavage suffers similar limitation due to the preference for NHEJ in DNA repair. Precise gene editing would appear currently to hold the most promise with the greatest initial frequency of gene correction but this method requires the development of unique reagents for each different mutation to be treated. Perhaps Wikipedia’s closing statement is prescient, ‘Gene therapy, while promising, requires a great deal more study.’

## References

[1] Fölling A Über ausscheidung von phenylbrenztraubensäure in den harn als stoffwechselanomalie in verbindung mit imbezillat. Hoppe-Seyl Z. 227, 169–176 (1934).

[2] Penrose L & Quastel JH Metabolic studies in phenylketonuria. Biochem. J 31(2), 266–274 (1937). 16746333 10.1042/bj0310266 PMC1266927

[3] Jervis GA Phenylpyruvic oligophrenia deficiency of phenylalanine-oxidizing system. Proc. Soc. Exp. Biol. Med 82(3), 514–515 (1953). 13047448

[4] Guthrie R & Susi A A simple phenylalanine method for detecting phenylketonuria in large populations of newborn infants. Pediatrics 32, 338–343 (1963). 14063511

[5] Dhondt JL Lessons from 30 years of selective screening for tetrahydrobiopterin deficiency. J. Inherit. Metab. Dis 33(Suppl 2), S219–S223 (2010). 10.1007/s10545-010-9091-9 20458544

[6] Anikster Y Biallelic mutations in DNAJC12 cause hyperphenylalaninemia, dystonia, and intellectual disability. Am. J. Hum. Genet 100(2), 257–266 (2017). 28132689 10.1016/j.ajhg.2017.01.002 PMC5294665

[7] Singh RH Recommendations for the nutrition management of phenylalanine hydroxylase deficiency. Genet. Med 16(2), 121–131 (2014). 24385075 10.1038/gim.2013.179 PMC3918542

[8] Vockley J Phenylalanine hydroxylase deficiency: diagnosis and management guideline. Genet. Med 16(2), 188–200 (2014). 24385074 10.1038/gim.2013.157

[9] National Institutes of Health Consensus Development, P., National Institutes of Health Consensus Development Conference Statement: phenylketonuria: screening and management, October 16–18, 2000. Pediatrics 108(4), 972–982 (2001). 11581453 10.1542/peds.108.4.972

[10] van Wegberg AMJ The complete European guidelines on phenylketonuria: diagnosis and treatment. Orphanet. J. Rare Dis 12(1), 162 (2017). 29025426 10.1186/s13023-017-0685-2 PMC5639803

[11] Levy HL Efficacy of sapropterin dihydrochloride (tetrahydrobiopterin, 6R-BH4) for reduction of phenylalanine concentration in patients with phenylketonuria: a phase III randomised placebo-controlled study. Lancet 370(9586), 504–510 (2007). 17693179 10.1016/S0140-6736(07)61234-3

[12] Zurfluh MR Molecular genetics of tetrahydrobiopterin-responsive phenylalanine hydroxylase deficiency. Hum. Mutat 29(1), 167–175 (2008). 17935162 10.1002/humu.20637

[13] Blau N & Scriver CR New approaches to treat PKU: how far are we? Mol. Genet. Metab 81(1), 1–2 (2004). 14728984 10.1016/j.ymgme.2003.09.011

[14] Burton BK The response of patients with phenylketonuria and elevated serum phenylalanine to treatment with oral sapropterin dihydrochloride (6R-tetrahydrobiopterin): a phase II, multicentre, open-label, screening study. J. Inherit. Metab. Dis 30(5), 700–707 (2007). 17846916 10.1007/s10545-007-0605-z

[15] Trefz FK Efficacy of sapropterin dihydrochloride in increasing phenylalanine tolerance in children with phenylketonuria: a phase III, randomized, double-blind, placebo-controlled study. J. Pediatr 154(5), 700–707 (2009). 19261295 10.1016/j.jpeds.2008.11.040

[16] Longo N Single-dose, subcutaneous recombinant phenylalanine ammonia lyase conjugated with polyethylene glycol in adult patients with phenylketonuria: an open-label, multicentre, phase 1 dose-escalation trial. Lancet 384(9937), 37–44 (2014). 24743000 10.1016/S0140-6736(13)61841-3 PMC4447208

[17] Harding CO Pegvaliase for the treatment of phenylketonuria: a pivotal, double-blind randomized discontinuation Phase 3 clinical trial. Mol. Genet. Metab 124(1), 20–26 (2018). 29628378 10.1016/j.ymgme.2018.03.003

[18] Longo N Long-term safety and efficacy of pegvaliase for the treatment of phenylketonuria in adults: combined phase 2 outcomes through PAL-003 extension study. Orphanet. J. Rare Dis 13(1), 108 (2018). 29973227 10.1186/s13023-018-0858-7 PMC6031112

[19] Thomas J Pegvaliase for the treatment of phenylketonuria: results of a long-term phase 3 clinical trial program (PRISM). Mol. Genet. Metab 124(1), 27–38 (2018). 29653686 10.1016/j.ymgme.2018.03.006

[20] Bickel H, Gerrard J & Hickmans EM Influence of phenylalanine intake on phenylketonuria. Lancet 265(6790), 812–813 (1953). 13098090 10.1016/s0140-6736(53)90473-5

[21] Armstrong MD & Tyler FH Studies on phenylketonuria. I. Restricted phenylalanine intake in phenylketonuria. J. Clin. Invest 34(4), 565–580 (1955). 14367510 10.1172/JCI103105 PMC438662

[22] Woolf LI, Griffiths R & Moncrieff A Treatment of phenylketonuria with a diet low in phenylalanine. Br. Med. J 1(4905), 57–64 (1955). 13219342 10.1136/bmj.1.4905.57 PMC2060789

[23] Blainey JD & Gulliford R Phenylalanine-restricted diets in the treatment of phenylketonuria. Arch. Dis. Child 31(160), 452–466 (1956). 13395572 10.1136/adc.31.160.452 PMC2012011

[24] Braude H Phenylketonuria; a case report in a European child treated with a diet low in phenylalanine. S. Afr. Med. J 30(4), 83–85 (1956). 13311579

[25] Horner FA & Streamer CW Effect of a phenylalanine-restricted diet on patients with phenylketonuria; clinical observations in three cases. J. Am. Med. Assoc 161(17), 1628–1630 (1956). 13345635 10.1001/jama.1956.62970170004005b

[26] Woolf LI The dietary treatment of phenylketonuria. Arch. Dis. Child 33(167), 31–45 (1958). 13509741 10.1136/adc.33.167.31 PMC2012184

[27] Williamson M, Dobson JC & Koch R Collaborative study of children treated for phenylketonuria: study design. Pediatrics 60(6), 815–821 (1977). 600593

[28] Azen CG Intellectual development in 12-year-old children treated for phenylketonuria. Am. J. Dis. Child 145(1), 35–39 (1991). 1985428 10.1001/archpedi.1991.02160010037012

[29] Koch R Phenylketonuria in adulthood: a collaborative study. J. Inherit. Metab. Dis 25(5), 333–346 (2002). 12408183 10.1023/a:1020158631102

[30] Walter JH & White FJ Blood phenylalanine control in adolescents with phenylketonuria. Int. J. Adolesc. Med. Health 16(1), 41–45 (2004). 15148857 10.1515/ijamh.2004.16.1.41

[31] Jurecki ER Adherence to clinic recommendations among patients with phenylketonuria in the United States. Mol. Genet. Metab 120(3), 190–197 (2017). 28162992 10.1016/j.ymgme.2017.01.001

[32] Bilder DA Neuropsychiatric comorbidities in adults with phenylketonuria: a retrospective cohort study. Mol. Genet. Metab 121(1), 1–8 (2017). 28285739 10.1016/j.ymgme.2017.03.002

[33] Manti F Psychiatric disorders in adolescent and young adult patients with phenylketonuria. Mol. Genet. Metab 117(1), 12–18 (2016). 26655635 10.1016/j.ymgme.2015.11.006

[34] Bilder DA Systematic review and meta-analysis of neuropsychiatric symptoms and executive functioning in adults with phenylketonuria. Dev. Neuropsychol 41(4), 245–260 (2016). 27805419 10.1080/87565641.2016.1243109 PMC5152552

[35] Antenor-Dorsey JA White matter integrity and executive abilities in individuals with phenylketonuria. Mol. Genet. Metab 109(2), 125–131 (2013). 23608077 10.1016/j.ymgme.2013.03.020 PMC3678378

[36] White DA White matter integrity and executive abilities following treatment with tetrahydrobiopterin (BH4) in individuals with phenylketonuria. Mol. Genet. Metab 110(3), 213–217 (2013). 23928118 10.1016/j.ymgme.2013.07.010 PMC3832288

[37] Pietz J Neurological outcome in adult patients with early-treated phenylketonuria. Eur. J. Pediatr 157(10), 824–830 (1998). 9809823 10.1007/s004310050945

[38] Thompson AJ Neurological deterioration in young adults with phenylketonuria. Lancet 336, 602–605 (1990). 1975386 10.1016/0140-6736(90)93401-a

[39] Platt LD The international study of pregnancy outcome in women with maternal phenylketonuria: report of a 12-year study. Am. J. Obstet. Gynecol 182(2), 326–333 (2000). 10694332 10.1016/s0002-9378(00)70219-5

[40] Darling G Serum selenium levels in individuals on PKU diets. J. Inherit. Metab. Dis 15(5), 769–773 (1992). 1434516 10.1007/BF01800019

[41] Hanley WB Vitamin B12 deficiency in adolescents and young adults with phenylketonuria. Eur. J. Pediatr 155(Suppl 1), S145–S147 (1996). 8828632 10.1007/pl00014233

[42] Robinson M Increased risk of vitamin B12 deficiency in patients with phenylketonuria on an unrestricted or relaxed diet. J. Pediatr 136(4), 545–547 (2000). 10753257 10.1016/s0022-3476(00)90022-2

[43] Hennermann JB Chronic kidney disease in adolescent and adult patients with phenylketonuria. J. Inherit. Metab. Dis 36(5), 747–756 (2013). 23138985 10.1007/s10545-012-9548-0

[44] Adamczyk P Bone metabolism and the muscle-bone relationship in children, adolescents and young adults with phenylketonuria. J. Bone Miner. Metab 29(2), 236–244 (2011). 20706750 10.1007/s00774-010-0216-x

[45] Choukair D Analysis of the functional muscle-bone unit of the forearm in patients with phenylketonuria by peripheral quantitative computed tomography. J. Inherit. Metab. Dis 40(2), 219–226 (2017). 27878409 10.1007/s10545-016-0002-6

[46] Brown CS & Lichter-Konecki U Phenylketonuria (PKU): a problem solved? Mol. Genet. Metab. Rep 6, 8–12 (2016). 27014571 10.1016/j.ymgmr.2015.12.004 PMC4789336

[47] Lichter-Konecki U, Hipke CM & Konecki DS Human phenylalanine hydroxylase gene expression in kidney and other nonhepatic tissues. Mol. Genet. Metab 67(4), 308–16 (1999). 10444341 10.1006/mgme.1999.2880

[48] Vajro P Correction of phenylketonuria after liver transplantation in a child with cirrhosis. N. Engl. J. Med 329(5), 363 (1993). 10.1056/NEJM199307293290517 8321274

[49] McDonald JD Pahhph-5: a mouse mutant deficient in phenylalanine hydroxylase. Proc. Natl. Acad. Sci. USA 87(5), 1965–1967 (1990). 2308957 10.1073/pnas.87.5.1965 PMC53605

[50] McDonald JD & Charlton CK Characterization of mutations at the mouse phenylalanine hydroxylase locus. Genomics 39(3), 402–405 (1997). 9119379 10.1006/geno.1996.4508

[51] Hamman K Low therapeutic threshold for hepatocyte replacement in murine phenylketonuria. Mol. Ther 12(2), 337–44 (2005). 16043102 10.1016/j.ymthe.2005.03.025 PMC2694052

[52] Hamman KJ, Winn SR & Harding CO Hepatocytes from wild-type or heterozygous donors are equally effective in achieving successful therapeutic liver repopulation in murine phenylketonuria (PKU). Mol. Genet. Metab 104(3), 235–40 (2011). 21917493 10.1016/j.ymgme.2011.07.027 PMC3219060

[53] Laconi E & Laconi S Principles of hepatocyte repopulation. Semin. Cell. Dev. Biol 13(6), 433–8 (2002). 12468244 10.1016/s1084952102001313

[54] Fox IJ Treatment of the Crigler-Najjar syndrome type I with hepatocyte transplantation. N. Engl. J. Med 338(20), 1422–6 (1998). 9580649 10.1056/NEJM199805143382004

[55] Horslen SP Isolated hepatocyte transplantation in an infant with a severe urea cycle disorder. Pediatrics 111(6 Pt 1), 1262–7 (2003). 12777539 10.1542/peds.111.6.1262

[56] Reyes J The use of cultured hepatocyte infusion via the portal vein for the treatment of ornithine transcarbamoylase deficiency by transplantation of enzymatically competent ABO/Rh-matched cells. Hepatology 24, 308A (1996).

[57] Meyburg J Human heterologous liver cells transiently improve hyperammonemia and ureagenesis in individuals with severe urea cycle disorders. J. Inherit. Metab. Dis 41(1), 81–90 (2018). 29027067 10.1007/s10545-017-0097-4

[58] Fanti M Differentiation of amniotic epithelial cells into various liver cell types and potential therapeutic applications. Placenta 59, 139–45 (2017). 28411944 10.1016/j.placenta.2017.03.020

[59] Grisch-Chan HM State-of-the-art 2019 on gene therapy for phenylketonuria. Hum. Gene Ther 30(10), 1274–83 (2019). 31364419 10.1089/hum.2019.111 PMC6763965

[60] Viecelli HM Treatment of phenylketonuria using minicircle-based naked-DNA gene transfer to murine liver. Hepatology 60(3), 1035–43 (2014). 24585515 10.1002/hep.27104 PMC4449723

[61] Fang B Gene therapy for phenylketonuria: phenotypic correction in a genetically deficient mouse model by adenovirus-mediated hepatic gene transfer. Gene Ther. 1(4), 247–54 (1994). 7584088

[62] Nagasaki Y Reversal of hypopigmentation in phenylketonuria mice by adenovirus-mediated gene transfer. Pediatr. Res 45(4 Pt 1), 465–73 (1999). 10203136 10.1203/00006450-199904010-00003

[63] Raper SE Fatal systemic inflammatory response syndrome in a ornithine transcarbamylase deficient patient following adenoviral gene transfer. Mol. Genet. Metab 80(1–2), 148–58 (2003). 14567964 10.1016/j.ymgme.2003.08.016

[64] https://clinicaltrials.gov/.

[65] Oh HJ Long-term enzymatic and phenotypic correction in the phenylketonuria mouse model by adeno-associated virus vector-mediated gene transfer. Pediatr. Res 56(2), 278–84 (2004). 15181195 10.1203/01.PDR.0000132837.29067.0E

[66] Mochizuki S Long-term correction of hyperpheylalaninemia by AAV-mediated gene transfer leads to behavioral recovery in phenylketonuria mice. Gene Ther. 11(13), 1081–6 (2004). 15057263 10.1038/sj.gt.3302262

[67] Embury J Hepatitis virus protein X-phenylalanine hydroxylase fusion proteins identified in PKU mice treated with AAV-WPRE vectors*. Gene Ther*. Mol. Biol 12, 69–76 (2008).

[68] Harding CO Complete correction of hyperphenylalaninemia following liver-directed, recombinant AAV2/8 vector-mediated gene therapy in murine phenylketonuria. Gene Ther. 13(5), 457–62 (2006). 16319949 10.1038/sj.gt.3302678 PMC2813194

[69] Ding Z, Georgiev P & Thony B Administration-route and gender-independent long-term therapeutic correction of phenylketonuria (PKU) in a mouse model by recombinant adeno-associated virus 8 pseudotyped vector-mediated gene transfer. Gene Ther. 13(7), 587–93 (2006). 16319947 10.1038/sj.gt.3302684

[70] Yagi H Complete restoration of phenylalanine oxidation in phenylketonuria mouse by a self-complementary adeno-associated virus vector. J. Gene Med 13(2), 114–22 (2011). 21322099 10.1002/jgm.1543

[71] Wang L Hepatic gene transfer in neonatal mice by adeno-associated virus serotype 8 vector. Hum. Gene Ther 23(5), 533–9 (2012). 22098408 10.1089/hum.2011.183 PMC3360497

[72] Cunningham SC AAV2/8-mediated correction of OTC deficiency is robust in adult but not neonatal *Spf*^*ash*^ mice. Mol. Ther 17(8), 1340–1346 (2009). 19384294 10.1038/mt.2009.88 PMC2835243

[73] Milani M Phagocytosis-shielded lentiviral vectors improve liver gene therapy in nonhuman primates. Sci. Transl. Med 11(493), pii: eaav7325 (2019). 10.1126/scitranslmed.aav7325 PMC7613847 31118293

[74] Hsu PD, Lander ES & Zhang F Development and applications of CRISPR-Cas9 for genome engineering. Cell 157(6), 1262–78 (2014). 24906146 10.1016/j.cell.2014.05.010 PMC4343198

[75] Cong L Multiplex genome engineering using CRIS-PR/Cas systems. Science 339(6121), 819–23 (2013). 23287718 10.1126/science.1231143 PMC3795411

[76] Mali P RNA-guided human genome engineering via Cas9. Science 339(6121), 823–6 (2013). 23287722 10.1126/science.1232033 PMC3712628

[77] Huai C CRISPR/Cas9-mediated somatic and germline gene correction to restore hemostasis in hemophilia B mice. Hum. Genet 136(7), 875–883 (2017). 28508290 10.1007/s00439-017-1801-z

[78] Yin H Genome editing with Cas9 in adult mice corrects a disease mutation and phenotype. Nat. Biotechnol 32(6), 551–3 (2014). 24681508 10.1038/nbt.2884 PMC4157757

[79] Ohmori T CRISPR/Cas9-mediated genome editing via postnatal administration of AAV vector cures haemophilia B mice. Sci. Rep 7(1), 4159 (2017). 28646206 10.1038/s41598-017-04625-5 PMC5482879

[80] Yang Y A dual AAV system enables the Cas9-mediated correction of a metabolic liver disease in newborn mice. Nat. Biotechnol 34(3), 334–8 (2016). 26829317 10.1038/nbt.3469 PMC4786489

[81] Richards DY AAV-mediated CRISPR/Cas9 gene editing in murine phenylketonuria. Mol. Ther. Methods Clin. Dev, in press (2020). 10.1016/j.omtm.2019.12.004 PMC6962637 31970201

[82] Komor AC Programmable editing of a target base in genomic DNA without double-stranded DNA cleavage. Nature 533(7603), 420–4 (2016). 27096365 10.1038/nature17946 PMC4873371

[83] Gaudelli NM Programmable base editing of A*T to G*C in genomic DNA without DNA cleavage. Nature 551(7681), 464–71 (2017). 29160308 10.1038/nature24644 PMC5726555

[84] Villiger L Treatment of a metabolic liver disease by in vivo genome base editing in adult mice. Nat. Med 24(10), 1519–25 (2018). 30297904 10.1038/s41591-018-0209-1

[85] Anzalone AV Search-and-replace genome editing without double-strand breaks or donor DNA. Nature, doi: 10.1038/s41586-019-1711-4 (2019). PMC6907074 31634902

